# Does neuroinflammation turn on the flame in Alzheimer's disease? Focus on astrocytes

**DOI:** 10.3389/fnins.2015.00259

**Published:** 2015-07-29

**Authors:** Luca Steardo, Maria R. Bronzuoli, Aniello Iacomino, Giuseppe Esposito, Luca Steardo, Caterina Scuderi

**Affiliations:** ^1^Department of Psychiatry, University of Naples SUNNaples, Italy; ^2^Department of Physiology and Pharmacology “Vittorio Erspamer”, Sapienza University of RomeRome, Italy; ^3^Faculty of Psychology, University of Rome “G. Marconi”Rome, Italy

**Keywords:** glia, astrocyte, neuroinflammation, neurodegeneration, Alzheimer's disease

## Abstract

Data from animal models and Alzheimer's disease (AD) subjects provide clear evidence for an activation of inflammatory pathways during the pathogenetic course of such illness. Biochemical and neuropathological studies highlighted an important cause/effect relationship between inflammation and AD progression, revealing a wide range of genetic, cellular, and molecular changes associated with the pathology. In this context, glial cells have been proved to exert a crucial role. These cells, in fact, undergo important morphological and functional changes and are now considered to be involved in the onset and progression of AD. In particular, astrocytes respond quickly to pathology with changes that have been increasingly recognized as a continuum, with potentially beneficial and/or negative consequences. Although it is now clear that activated astrocytes trigger the neuroinflammatory process, however, the precise mechanisms have not been completely elucidated. Neuroinflammation is certainly a multi-faceted and complex phenomenon and, especially in the early stages, exerts a reparative intent. However, for reasons not yet all well known, this process goes beyond the physiologic control and contributes to the exacerbation of the damage. Here we scrutinize some evidence supporting the role of astrocytes in the neuroinflammatory process and the possibility that these cells could be considered a promising target for future AD therapies.

## Inflammation in Alzheimer's disease

Alzheimer's disease (AD) is a neurodegenerative disorder characterized by memory loss and significant cognitive decline with impaired activities of daily living (Goedert and Spillantini, 2006). Despite all scientific efforts, medications currently used provide only modest and transient benefits to a subset of patients and effective pharmacotherapeutic options are lacking. Histopathologically, the hallmarks of AD are extracellular deposits of beta amyloid (Aβ) fibrils in senile plaques (SP) and intraneuronal neurofibrillary tangles (NFT). NFT are filamentous inclusions composed of hyperphosphorylated forms of the microtubule-associated protein tau (Blennow et al., 2006). Aβ results from the abnormal proteolytic cleavage of amyloid-precursor protein (APP) by the sequential action of beta- and gamma-secretase. The widely accepted Aβ cascade hypothesis suggests that this peptide is the direct cause of neurodegeneration, synaptic loss, and cognitive decline in AD (Selkoe, 1996). Even if several biochemical mechanisms have been proposed, including production of reactive oxygen species, disruption of calcium homeostasis, activation of Wnt pathway, excitotoxicity, activation of apoptotic pathways, neuronal degeneration, and neurotransmitter deficits, the precise role of abnormal protein aggregates in the pathogenesis of AD remains to be clarified (Huang and Jiang, 2009; Welsh-Bohmer and White, 2009; Querfurth and LaFerla, 2010). Interestingly, data from animal models and human autopsies revealed that both SP and NFT are co-localized with activated glial cells, suggesting that reactive gliosis might exert a key pathogenetic role in AD (Craft et al., 2006). Aβ peptides, probably through the main involvement of non-neuronal cells, promote neuroinflammation, accounting for the synthesis of different cytokines, and pro-inflammatory mediators (Mrak and Griffin, 2001; Tuppo and Arias, 2005). Recent evidence assigns to astrocyte dysfunctions a critical role in aging and in several neurodegenerative diseases, including AD. It is now clear that astrocytes are essential in the control of cerebral homeostasis. However, several brain injuries, including Aβ deposition, modify their physiological functioning and astrocyte acquire a reactive phenotype (Verkhratsky et al., 2014). Activation of these cells is fundamentally a protective response aimed at removing injurious stimuli. However, uncontrolled and prolonged activation goes beyond physiological control and detrimental effects override the beneficial ones. In this condition, astrocytes foster neuroinflammatory response, accounting for the synthesis of different cytokines and pro-inflammatory mediators. After their release, pro-inflammatory signal molecules act, in an autocrine way, to self perpetuate reactive gliosis and, in a paracrine way, to kill neighboring neurons expanding the neuropathological damage (Mrak and Griffin, 2001; Pekny et al., 2014).

In this context, it is important to highlight that inflammatory process, once initiated, may contribute independently to neural dysfunction and cell death, thereby establishing a self-fostering vicious cycle, by which the inflammation represents a substantial cause of further neurodegeneration (Block and Hong, 2005; Glass et al., 2010). Therefore, it is reasonable to assume that early combination of neuroprotective and anti-inflammatory treatments aimed at restoring astrocyte functions may represent an appropriate approach to treat AD, whereas the treatment of only one pathological process might even worsen the others (Scuderi et al., 2014a).

A growing body of evidence shows the remarkable complexity of inflammatory mechanisms in AD and how these mechanisms are often driven by alterations of glial cells functioning. Such complexity encourages to perform additional investigations in order to better clarify the mechanisms involved with the aim to develop appropriate therapeutics.

Can astrocytes become the target for new drugs? In the last decade, the neuron-centric vision of neuropsychiatric diseases has undergone considerable changes. Indeed, it is now clear that the non-neuronal cells could be involved in the pathogenesis and progression of many diseases due to their important and active roles exerted in the brain physiological and pathological conditions. Therefore, these cells could not be considered simple co-stars in the drama of neuropsychiatric disorders, including neurodegeneration.

Glial cells are non-excitable cells of the central nervous system (CNS). These cells are a highly heterogeneous population, responsible for many important brain functions (Doens and Fernández, 2014; Öngür et al., 2014; Hol and Pekny, 2015; Rodríguez-Arellano et al., 2015). The oligodendrocytes, for example, envelop axons with myelin and provide neurons with homeostatic support, and progenitor cells expressing proteoglycan NG2 most likely represent a reservoir of myelinating cells (Fröhlich et al., 2011; Walhovd et al., 2014). The microglial cells are regarded to be the innate immune cells in the CNS. They get activated in response to any type of brain injury, producing a wide range of pro- or anti-inflammatory mediators (Gundersen et al., 2015). Astrocytes are definitely the most abundant and heterogeneous type of glial cells in the CNS. Indeed, their morphology differs largely depending on their development stage, subtype, and localization. For example, the most abundant astrocytes of the gray matter are the protoplasmic ones, which exhibit short branches, whereas the fibrous astrocytes are present in the white matter and exhibit long unbranched processes (Verkhratsky and Parpura, 2015). It is well-proved that astrocytes control the brain homeostasis and allow neurons to functioning; they are an essential neuro-supportive cell type in brain, responsible for a massive number of homeostatic tasks in the CNS (Verkhratsky and Butt, 2013). Indeed, astrocytes finely control the environment by regulating pH, ion homeostasis, blood flow, and modulate oxidative stress (Deitmer and Rose, 1996; Wilson, 1997; Iadecola and Nedergaard, 2007; Obara et al., 2008). In addition, these cells importantly contribute to synaptogenesis and dynamically modulate information processing and signal transmission, regulate neural and synaptic plasticity, and provide trophic and metabolic support to neurons (Perea et al., 2009; Sofroniew, 2014). On the basis of all these considerations, it is reasonable to assume that alterations in some of these important neuro-supportive functions can result in injurious consequences for the brain (Bélanger and Magistretti, 2009).

It has been well established that brain insults trigger a specific astroglial reaction represented by a complex morphofunctional remodeling (Sun and Jakobs, 2012). Astrocytes, together with microglia, are the cellular component of the resident innate immune system in the CNS. They act as crucial effectors of the neuroinflammatory response (Ransohoff and Brown, 2012). Indeed, astrocytes rapidly act in response to pathology undergoing important changes in their morphology and functioning (Sofroniew and Vinters, 2010; Scuderi et al., 2013; Rossi, 2015). This reactive state starts with the intention to control and remove the brain damage, however it has deleterious consequences. In fact, reactive gliosis is a self-perpetuating process which, at the end, exacerbates the injury and, on another hand it represents a non-physiologic state in which astrocytes lose their helpful properties. These events are particularly patent in AD brain and Alzheimer himself was able to recognize, in autopsied specimens, a marked activation of astroglial cells and described a manifest inflammatory status (Alzheimer, 1907). Data from both humans and animal models of AD have demonstrated that reactive astrocytes co-localize with SP and NFT (Lukiw and Bazan, 2000; Armstrong, 2009; Rodríguez et al., 2009) suggesting that the primary aim of this peculiar localization is probably the creation of a barrier between healthy and injured tissue.

Additionally, accumulating data shows the relevant role of astrocytes in Aβ pathogenesis of AD. Numerous debates have discussed whether astrocytes uptake Aβ from the extracellular space or they could synthesize Aβ *de novo*.

Astrocytes possess tools to internalize and metabolize Aβ *in vivo*. They express the receptor for advanced glycation end product (RAGE) which binds Aβ, and possess a complex apparatus able to take up Aβ (Wyss-Coray et al., 2003). So, the notion that astrocytes phagocytose Aβ from the extracellular space is reinforced by a number of investigations, which proved that Aβ was taken up by astrocytes for lysosomal degradation in order to maintain Aβ homeostasis (Funato et al., 1998). It has been postulated that this complex machinery makes astrocytes the preferential type of glial cells liable to remove Aβ (Nagele et al., 2003; Wyss-Coray et al., 2003; Koistinaho et al., 2004). In other studies, a lower responsiveness to Aβ in human astrocytes from AD cases was reported, suggesting that SP formation in the extracellular space might be subsequent to insufficient clearance of Aβ by astrocytes (Mulder et al., 2012). APP and BACE1 overexpression in astrocytes may result in Aβ synthesis *de novo* (Zhao et al., 2011). It was reported an enhanced BACE1 production in reactive astrocytes, supporting the notion that Aβ accumulation in the extracellular space could be secondary to its production and release by activated astrocytes. Therefore, based upon this evidence it is possible to assert that astrocytes may have a dual role in either clearing and producing Aβ.

On the other side, exposure to Aβ causes deleterious consequences on astrocyte functioning. Indeed, these cells alter their morphology with a marked increase in the expression of the glial fibrillary acidic protein (GFAP), a recognized marker of astrocyte reactivity (Chow et al., 2010; Scuderi et al., 2014a). In parallel, Aβ challenge provokes alterations of calcium homeostasis, energetic modification, and degeneration of co-cultured neurons (Malchiodi-Albedi et al., 2001; Abramov et al., 2004; Chow et al., 2010; Scuderi et al., 2011, 2012). Lastly, Aβ insult also results in increased oxidative and nitrosative stress (Frank-Cannon et al., 2009). It has been demonstrated that Aβ accumulation in SP causes over-expression of a number of inflammation-related factors, such as nitric oxide (NO) interleukin (IL)-1α, IL-1β, and tumor necrosis factor (TNF)-α (Li et al., 2003; Esposito et al., 2006). An increasing body of evidence demonstrates that NO represents one of the major effectors of neuronal cell death through mitochondrial depolarization (Solenski et al., 2003), while IL-1β enhances activation of caspase-3, an enzyme implicated in hippocampal neuron apoptotic death in aged rats (Lynch and Lynch, 2002). Similarly, TNF-α has been demonstrated to be involved in neuronal apoptosis induced by Aβ, activating the caspase-cascade, through the interaction with its specific type 1 receptor (Li et al., 2004a).

Transgenic APP_(SWE)_ (Tg2576) mice, which over-express the human APP gene, show high levels of interferon-γ and IL-12 mRNA, as well as their protein production, and such an increase was found in the reactive astrocytes surrounding Aβ deposits (Abbas et al., 2002). Some authors demonstrated that some of these effects may be due to alterations in astrocyte transcription factors. For example, the CCAAT-enhancer binding protein (C/EBP) family of transcription factors, which regulates or co-regulates a wide range of inflammatory mediators, is considerably higher in reactive astrocytes surrounding Aβ deposits of AD cortex in comparison with non-AD age matched controls (Li et al., 2004b).

In addition it was demonstrated that astrocytic NF-κB is activated after Aβ exposure, causing an increased expression and release of a variety of inflammatory molecules including IL-1β and IL-6 (Bales et al., 1998). Moreover, the activation of NF-κB in astrocytes is also responsible in mediating the inflammatory process through the expression of adhesion molecules and chemokines which allow the invasion by peripheral leukocytes (Moynagh, 2005). Although the impact of astroglial inactivation of NF-κB has not been established in AD mouse models, recent evidence demonstrates that blockage of NF-κB transcriptional activity in astrocytes can extensively reduce inflammation and improve recovery, thus suggesting that inhibition of NF-κB in astrocytes may be regarded as a potential therapy for AD (Medeiros and LaFerla, 2013).

Among the many released mediators, S100B represents a key factor during neuroinflammation. This small peptide is almost exclusively produced by astrocytes and, under physiological conditions, it is a neurotrophin responsible for survival, development, and function of neurons (Donato, 2003). In AD, but also in Down's syndrome (whose subjects often develop a precocious AD-like dementia), in Parkinson's disease, and in subjects with severe brain trauma, S100B is over-expressed and its levels correlate with the progression of the pathology (Mrak et al., 1996; Sheng et al., 2000). A similar correlation is detectable in the brain of APPV717F, transgenic mice which over-express a human APP minigene encoding a familial AD mutation. In APPV717F is evident an age-related increase in tissue levels of both APP and S100B mRNA and such an increase precedes the appearance of neuritic plaques (Sheng et al., 2000). Lastly, by using S100B-overexpressing transgenic and S100B knockout mice intracerebroventricular injected with human oligomeric Aβ_1−42_, it was established a relationship between S100B levels and susceptibility to AD-relevant neuroinflammation and neuronal dysfunction. Indeed, after Aβ infusion inflammatory reaction in S100B-overexpressing transgenic mice was markedly higher than in knock-out or wild-type mice (Craft et al., 2005).

In addition, electrophysiological and behavioral investigations demonstrated that S100B affects long-term synaptic plasticity and impairs cognitive tasks (Gerlai, 1998; Nishiyama et al., 2002). All these considerations emphasize the assumption that S100B up-regulation may result in deleterious consequences and that early targeting of this protein could be beneficial in preventing or slowing AD progression. Finally, to further support the assumption that a protracted reactive gliosis is importantly implicated in the pathobiology of AD, proton resonance spectroscopy consistently provided evidence of a significant increase of myoinositol (an astroglial marker) in brain areas of both mild cognitive impairment (MCI) and AD patients. Such an increase, according to some studies, have been reported to correlate with the progression of pathology (Kantarci et al., 2008; Tumati et al., 2013).

Given the complex heterogeneity of pathological changes occurring in AD, scientists have to increase their efforts in preclinical and clinical research to identify new therapeutics.

In the last 15 years, growing data highlighted the important genetic, epigenetic, and molecular changes occurring in AD. These studies provided the evidence for another important actor in the drama of this neurological disorder: the marked neuroinflammation. This process is really more complex than it could appear at first glance. Indeed, neuroinflammation is generated and orchestrated by glial cells. In particular, microglia and astrocytes actively participate in mediating the so-called reactive gliosis and, by interacting with neurons, they take part in the onset and progression of the disease. Despite its recognized crucial role in AD, the precise molecular pathways of neuroinflammation remain unclear. It is mandatory to characterize these fine mechanisms, as well as to define time, brain regions, and cell type involved in mediating this multifaceted process. Although epidemiological studies shed light on a possible benefit of long-term use of nonsteroidal anti-inflammatory drugs (NSAIDs), however it could be wrongful and simplistic to suggest these drugs as the AD treatment, because it is erroneous to regard AD as a mere neuroinflammatory disease. In addition, many randomized clinical trials, designed to test the efficacy of a long-term use of NSAIDs in AD, failed to demonstrate that these drugs slow down the progression of disease or improve the symptoms in patients with early or moderate AD (Thal et al., 2005; Breitner et al., 2009; Szekely and Zandi, 2010). The reasons of these failures could be numerous; for example, the peculiar nature of the inflammatory process, the advanced state of the disease, and the dosing regimens of the trials. In this context it is also important to mention as a study demonstrated that autopsied specimens from very old humans with cognitively normal functions showed higher expression of complement factors and several immune molecules than brains from age-matched AD patients or younger people (Katsel et al., 2009). This evidence seems to suggest that molecules, typically seen as deleterious, exert simultaneously multiple roles. For example, Cyclooxygenase(COX)-2 is implicated in both physiological and pathological processes. This renders the understanding of the role of this enzyme in the brain somewhat tricky (Hoozemans et al., 2001). Unlike COX-1, which is primarily localized in human microglial cells, COX-2 is mainly expressed in pyramidal neurons where it may be involved in learning and memory functions. The importance to attenuate astroglial inflammation in AD has been also demonstrated by adeno-associated virus-driven suppression of the astrocyte reaction in APP/PS1 mice, which revealed improved cognition, reduced astrogliosis, and decreased Aβ concentrations (Furman et al., 2012).

A further important aspect that requires to be clarified concerns the temporal relationships between Aβ accumulation and glial activation. Human studies have revealed a parallel increase in activation of both astrocyte and microglia and AD progression. This occurs independently from the Aβ deposition, suggesting that gliosis could *per se* induce neurodegeneration (Serrano-Pozo et al., 2011). Likewise, using a multitracer PET investigation in patients with MCI and AD, astrocytosis was proved to be an early phenomenon in AD progression (Carter et al., 2012).

Therefore, the dominant view that astrocytes respond secondarily to neuronal damage could be contradicted by growing evidence supporting an active role of these cells as primary players in several mechanisms related to the pathophysiology of AD.

The interplay between dysfunctional astroglial cells and adjacent neurons can start a sequence of events that results in neuronal damage and the appearance of the characteristic AD hallmarks. Moreover, since astrocytes exert an important role in maintaining synaptic homeostasis, astrocyte malfunctioning may negatively influence the efficiency of neuronal circuits.

## Conclusion

It is widely accepted that protracted astrogliosis is a prominent component of AD pathology and might represent an attractive therapeutic target. However, since reactive gliosis is now identified as a complex process with both helpful and detrimental aspects, it will be crucial an understanding that is not limited to phenomenological descriptions but also able to clarify the relevant disease-modifying pathways. The challenge for future studies, therefore, will be to identify tools capable of selectively prevent the over-activation of these cells and pathways without interfering with their physiological and beneficial properties. Currently a fair number of compounds seems to be able to restore a normal astrocyte functioning in AD models, serving as novel potential tools in the treatment of AD pathology (Scuderi et al., 2014a,b).

## Author contributions

MRB, AI and LS Jr. drafted the manuscript. CS, GE, and LS revised and approved the final version of the manuscript. All authors ensure that questions related to the accuracy or integrity of any part of the work were appropriately investigated and resolved.

### Conflict of interest statement

The authors declare that the research was conducted in the absence of any commercial or financial relationships that could be construed as a potential conflict of interest.
